# (*E*)-*N*′-(5-Chloro-2-hy­droxy­benzyl­idene)-2,4-dihy­droxybenzohydrazide methanol monosolvate

**DOI:** 10.1107/S1600536812009178

**Published:** 2012-03-07

**Authors:** Kun Li

**Affiliations:** a College of Life Sciences, Liaoning Normal University, Dalian 116029, People’s Republic of China

## Abstract

In the title compound, C_14_H_11_ClN_2_O_4_·CH_3_OH, the mol­ecule adopts an *E* conformation about the C=N bond. The compound is in the enamine–keto form. The two terminal benzene rings make a dihedral angle of 10.53 (9)°. Intra-mol­ecular O—H⋯O and O—H⋯N hydrogen bonding stabilizes the mol­ecular structure. In the crystal, O—H⋯O hydrogen bonds link the mol­ecules, forming chains running along the *b* axis.

## Related literature

For general background to the bioactivity of Schiff bases in the pharmaceutical and agrochemical fields, see: Bernardino *et al.* (2006 ▶); Zhang *et al.* (2008 ▶). For related compounds, see: Huang *et al.* (2008 ▶); Zhang *et al.* (2007 ▶). For a related structure, see: Deng *et al.* (2009 ▶).
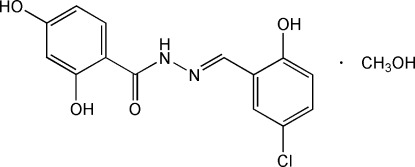


## Experimental

### 

#### Crystal data

C_14_H_11_ClN_2_O_4_·CH_4_O
*M* *_r_* = 338.74
Monoclinic, 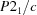

*a* = 7.5438 (11) Å
*b* = 13.1623 (19) Å
*c* = 15.903 (2) Å
β = 103.251 (3)°
*V* = 1537.0 (4) Å^3^
*Z* = 4
Mo *K*α radiation
μ = 0.28 mm^−1^
*T* = 296 K
0.20 × 0.12 × 0.10 mm

#### Data collection

Brucker SMART 1000 CCD diffractometer
14496 measured reflections
3808 independent reflections
2407 reflections with *I* > 2σ(*I*)
*R*_int_ = 0.038

#### Refinement

*R*[*F* ^2^ > 2σ(*F* ^2^)] = 0.044
*wR*(*F* ^2^) = 0.119
*S* = 1.01
3808 reflections
209 parameters
H-atom parameters constrained
Δρ_max_ = 0.18 e Å^−3^
Δρ_min_ = −0.29 e Å^−3^

### 

Data collection: *SMART* (Bruker, 2001 ▶); cell refinement: *SAINT-Plus* (Bruker, 2003 ▶); data reduction: *SAINT-Plus*; program(s) used to solve structure: *SHELXTL* (Sheldrick, 2008 ▶); program(s) used to refine structure: *SHELXTL*; molecular graphics: *SHELXTL*; software used to prepare material for publication: *SHELXTL*.

## Supplementary Material

Crystal structure: contains datablock(s) I, global. DOI: 10.1107/S1600536812009178/xu5474sup1.cif

Structure factors: contains datablock(s) I. DOI: 10.1107/S1600536812009178/xu5474Isup2.hkl

Supplementary material file. DOI: 10.1107/S1600536812009178/xu5474Isup3.cml

Additional supplementary materials:  crystallographic information; 3D view; checkCIF report

## Figures and Tables

**Table 1:** Hydrogen-bond geometry (Å, °)

*D*—H⋯*A*	*D*—H	H⋯*A*	*D*⋯*A*	*D*—H⋯*A*
N2—H2⋯O5	0.86	2.01	2.833 (2)	159
O1—H1*A*⋯N1	0.82	1.81	2.5333 (19)	146
O3—H3⋯O2	0.82	1.77	2.5030 (18)	148
O4—H4*A*⋯O1^i^	0.82	2.00	2.7401 (19)	151
O5—H5*A*⋯O3^i^	0.82	2.03	2.8346 (19)	168

Symmetry code: (i) 
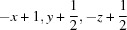
.

**Fig. 1.**
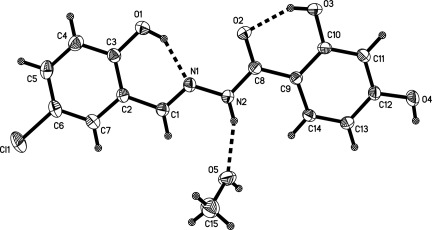
The molecular structure of (I) (thermal ellipsoids are shown at 30% probability levels).

**Fig. 2.**
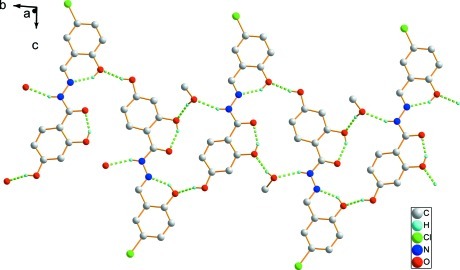
A diagram showing hydrogen bonds of the title compound.

## supplementary crystallographic information

### Comment

Schiff bases have attracted much attention due to their diverse range of bioactivities in pharmaceutical and agrochemical field (Bernardino *et al.*, 2006; Zhang *et al.*, 2008). In order to expand this field, we now report the synthsis and structure of the title compound, (I), (Fig. 1).

In the title compound, the bond length of 1.274 (2) Å between atoms C(1) and N(1) is similar to those observed in other Schiff basess (Deng *et al.*, 2009; Huang *et al.*., 2008; Zhang *et al.*., 2007), indicating it is a double bond. The bond length of C(8)–N(2), 1.357 (2) Å, is intermediate between C–N and C=N bonds due to the conjugation effects in the molecule. The mean planes of the two benzene rings make a dihedral angle of 10.53 (3)°. As expected, the molecule adopts a *trans* configuration about the C=N double bond. As expected, the molecule adopts a *trans* configuration about the C=N double bond. The torsion angles C(9)–C(8)–N(2)–N(1), O(2)–C(8)–N(2)–N(1), C(2)–C(1)–N(1)–N(2)–and C(1)–N(1)–N(2)–C(8) are -174.10 (14), 5.6 (2), -178.63 (14) and 177.27 (15)°, respectively. Three Intramolecular hydrogen bonds are observed in the molecular structure. The lattice methanol and hydroxyl group of the Schiff base in the crystal are linked to the Schiff base moieties through intermolecular N–H···O, O–H···O hydrogen bonds (Table 1, Figs. 1 and2). The title compound extends further to its final two-dimensional network through intermolecular N—H···O, O–H···O hydrogen bonds which interlink molecules stabilize the structure. (Table 1, Fig 2).

### Experimental

5-chloro-2-hydroxybenzaldehyde (0.1 mmol, 15.6 mg) and 2,4-Dihydroxybenzhydrazide (0.1 mmol, 16.8 mg) were dissolved in a methanol solution (10 ml). The mixture was stirred at room temperature for 1 h and filtered. After keeping the filtrate in air for three days, yellow rod-like crystals were formed.

### Refinement

H atoms were placed in geometrically idealized positions and allowed to ride on their parent atoms, O—H = 0.82, N—H = 0.86, C—H = 0.93–0.96 Å, with *U*_iso_(H) = 1.5U_eq_(C,O) for methyl H and hydroxyl H atoms, and 1.2U_eq_(C,N) for the others.

### Crystal data

**Table:**

C_14_H_11_ClN_2_O_4_·CH_4_O	*F*(000) = 704
*M**_r_* = 338.74	*D*_x_ = 1.464 Mg m^−^^3^
Monoclinic, *P*2_1_/*c*	Mo *K*α radiation, λ = 0.71073 Å
Hall symbol: -P 2ybc	Cell parameters from 2195 reflections
*a* = 7.5438 (11) Å	θ = 3.1–26.4°
*b* = 13.1623 (19) Å	µ = 0.28 mm^−^^1^
*c* = 15.903 (2) Å	*T* = 296 K
β = 103.251 (3)°	Rod, yellow
*V* = 1537.0 (4) Å^3^	0.20 × 0.12 × 0.10 mm
*Z* = 4	

### Data collection

**Table:**

Brucker SMART 1000 CCD diffractometer	2407 reflections with *I* > 2σ(*I*)
Radiation source: fine-focus sealed tube	*R*_int_ = 0.038
Graphite monochromator	θ_max_ = 28.3°, θ_min_ = 2.0°
ω scans	*h* = −10→10
14496 measured reflections	*k* = −17→17
3808 independent reflections	*l* = −20→21

### Refinement

**Table:**

Refinement on *F*^2^	Primary atom site location: structure-invariant direct methods
Least-squares matrix: full	Secondary atom site location: difference Fourier map
*R*[*F*^2^ > 2σ(*F*^2^)] = 0.044	Hydrogen site location: inferred from neighbouring sites
*wR*(*F*^2^) = 0.119	H-atom parameters constrained
*S* = 1.01	*w* = 1/[σ^2^(*F*_o_^2^) + (0.0492*P*)^2^ + 0.2991*P*] where *P* = (*F*_o_^2^ + 2*F*_c_^2^)/3
3808 reflections	(Δ/σ)_max_ < 0.001
209 parameters	Δρ_max_ = 0.18 e Å^−^^3^
0 restraints	Δρ_min_ = −0.29 e Å^−^^3^

### Special details

**Table:**

Geometry. All e.s.d.'s (except the e.s.d. in the dihedral angle between two l.s. planes) are estimated using the full covariance matrix. The cell e.s.d.'s are taken into account individually in the estimation of e.s.d.'s in distances, angles and torsion angles; correlations between e.s.d.'s in cell parameters are only used when they are defined by crystal symmetry. An approximate (isotropic) treatment of cell e.s.d.'s is used for estimating e.s.d.'s involving l.s. planes.
Refinement. Refinement of *F*^2^ against ALL reflections. The weighted *R*-factor *wR* and goodness of fit *S* are based on *F*^2^, conventional *R*-factors *R* are based on *F*, with *F* set to zero for negative *F*^2^. The threshold expression of *F*^2^ > σ(*F*^2^) is used only for calculating *R*-factors(gt) *etc*. and is not relevant to the choice of reflections for refinement. *R*-factors based on *F*^2^ are statistically about twice as large as those based on *F*, and *R*- factors based on ALL data will be even larger.

### Fractional atomic coordinates and isotropic or equivalent isotropic displacement parameters (Å^2^)

**Table:**

	*x*	*y*	*z*	*U*_iso_*/*U*_eq_	
C1	0.2107 (2)	0.06032 (14)	−0.02905 (10)	0.0381 (4)	
H1	0.2103	0.1310	−0.0285	0.046*	
C2	0.0958 (2)	0.00413 (13)	−0.09970 (10)	0.0350 (4)	
C3	0.0877 (2)	−0.10217 (14)	−0.09981 (11)	0.0393 (4)	
C4	−0.0226 (3)	−0.15334 (16)	−0.16831 (13)	0.0504 (5)	
H4	−0.0283	−0.2239	−0.1679	0.060*	
C5	−0.1240 (3)	−0.09963 (17)	−0.23709 (13)	0.0529 (5)	
H5	−0.1971	−0.1341	−0.2833	0.063*	
C6	−0.1173 (3)	0.00494 (17)	−0.23758 (12)	0.0464 (5)	
C7	−0.0092 (2)	0.05704 (15)	−0.16963 (11)	0.0424 (4)	
H7	−0.0064	0.1277	−0.1704	0.051*	
C8	0.5164 (2)	−0.00088 (13)	0.16440 (11)	0.0361 (4)	
C9	0.6206 (2)	0.04621 (12)	0.24364 (10)	0.0335 (4)	
C10	0.7254 (2)	−0.01654 (12)	0.30783 (11)	0.0361 (4)	
C11	0.8242 (3)	0.02260 (14)	0.38451 (11)	0.0431 (4)	
H11	0.8948	−0.0201	0.4255	0.052*	
C12	0.8181 (3)	0.12552 (14)	0.40030 (11)	0.0412 (4)	
C13	0.7145 (3)	0.19012 (13)	0.33873 (11)	0.0419 (4)	
H13	0.7100	0.2594	0.3496	0.050*	
C14	0.6194 (2)	0.15036 (13)	0.26201 (11)	0.0386 (4)	
H14	0.5518	0.1939	0.2208	0.046*	
C15	0.4082 (4)	0.3243 (2)	−0.00103 (16)	0.0741 (7)	
H15A	0.3001	0.3588	−0.0311	0.111*	
H15B	0.4478	0.2781	−0.0397	0.111*	
H15C	0.5025	0.3732	0.0199	0.111*	
Cl1	−0.24386 (8)	0.07252 (5)	−0.32463 (4)	0.0732 (2)	
N1	0.31182 (19)	0.01011 (11)	0.03204 (8)	0.0374 (3)	
N2	0.4195 (2)	0.05913 (11)	0.10084 (9)	0.0390 (3)	
H2	0.4254	0.1243	0.1037	0.047*	
O1	0.18685 (19)	−0.15812 (10)	−0.03310 (8)	0.0525 (4)	
H1A	0.2464	−0.1199	0.0034	0.079*	
O2	0.51353 (19)	−0.09463 (9)	0.15379 (8)	0.0459 (3)	
O3	0.73109 (19)	−0.11901 (9)	0.29692 (8)	0.0478 (3)	
H3	0.6682	−0.1344	0.2494	0.072*	
O4	0.9180 (2)	0.15923 (11)	0.47749 (9)	0.0637 (4)	
H4A	0.9057	0.2209	0.4808	0.096*	
O5	0.3709 (2)	0.27020 (11)	0.06876 (10)	0.0668 (5)	
H5A	0.3405	0.3099	0.1026	0.100*	

### Atomic displacement parameters (Å^2^)

**Table:**

	*U*^11^	*U*^22^	*U*^33^	*U*^12^	*U*^13^	*U*^23^
C1	0.0412 (10)	0.0383 (9)	0.0348 (9)	0.0007 (8)	0.0088 (8)	−0.0010 (7)
C2	0.0332 (9)	0.0423 (10)	0.0307 (8)	−0.0003 (7)	0.0095 (7)	0.0008 (7)
C3	0.0393 (10)	0.0418 (10)	0.0367 (9)	−0.0017 (8)	0.0084 (8)	0.0029 (8)
C4	0.0501 (12)	0.0474 (11)	0.0518 (11)	−0.0101 (9)	0.0080 (10)	−0.0052 (9)
C5	0.0449 (12)	0.0689 (15)	0.0416 (10)	−0.0118 (10)	0.0030 (9)	−0.0095 (10)
C6	0.0369 (10)	0.0652 (13)	0.0350 (9)	0.0005 (9)	0.0039 (8)	0.0061 (9)
C7	0.0406 (10)	0.0473 (11)	0.0392 (9)	0.0026 (8)	0.0090 (8)	0.0055 (8)
C8	0.0392 (10)	0.0365 (10)	0.0334 (8)	−0.0019 (8)	0.0104 (7)	0.0019 (7)
C9	0.0364 (9)	0.0333 (9)	0.0310 (8)	−0.0008 (7)	0.0082 (7)	0.0012 (7)
C10	0.0417 (10)	0.0291 (9)	0.0375 (9)	0.0018 (7)	0.0095 (8)	0.0025 (7)
C11	0.0486 (11)	0.0393 (10)	0.0370 (9)	0.0080 (8)	0.0004 (8)	0.0031 (8)
C12	0.0420 (10)	0.0414 (10)	0.0360 (9)	0.0044 (8)	0.0004 (8)	−0.0053 (8)
C13	0.0493 (11)	0.0312 (9)	0.0425 (10)	0.0036 (8)	0.0049 (8)	−0.0052 (7)
C14	0.0443 (10)	0.0330 (9)	0.0363 (9)	0.0059 (8)	0.0049 (8)	0.0029 (7)
C15	0.0878 (19)	0.0742 (17)	0.0599 (15)	−0.0097 (14)	0.0158 (14)	0.0039 (12)
Cl1	0.0588 (4)	0.1009 (5)	0.0494 (3)	0.0050 (3)	−0.0089 (3)	0.0195 (3)
N1	0.0392 (8)	0.0417 (8)	0.0310 (7)	−0.0023 (7)	0.0073 (6)	−0.0023 (6)
N2	0.0483 (9)	0.0352 (8)	0.0309 (7)	−0.0009 (7)	0.0035 (6)	−0.0020 (6)
O1	0.0621 (9)	0.0389 (7)	0.0491 (8)	−0.0038 (6)	−0.0025 (7)	0.0075 (6)
O2	0.0591 (9)	0.0321 (7)	0.0428 (7)	−0.0057 (6)	0.0037 (6)	−0.0020 (5)
O3	0.0663 (9)	0.0286 (6)	0.0439 (7)	0.0034 (6)	0.0026 (6)	0.0018 (5)
O4	0.0773 (11)	0.0503 (8)	0.0474 (8)	0.0148 (7)	−0.0187 (7)	−0.0138 (6)
O5	0.1037 (13)	0.0446 (8)	0.0534 (8)	0.0176 (8)	0.0206 (9)	0.0007 (7)

### Geometric parameters (Å, º)

**Table:**

C1—N1	1.274 (2)	C10—O3	1.362 (2)
C1—C2	1.454 (2)	C10—C11	1.375 (2)
C1—H1	0.9300	C11—C12	1.380 (3)
C2—C7	1.395 (2)	C11—H11	0.9300
C2—C3	1.400 (3)	C12—O4	1.359 (2)
C3—O1	1.365 (2)	C12—C13	1.393 (2)
C3—C4	1.385 (3)	C13—C14	1.370 (2)
C4—C5	1.377 (3)	C13—H13	0.9300
C4—H4	0.9300	C14—H14	0.9300
C5—C6	1.377 (3)	C15—O5	1.401 (3)
C5—H5	0.9300	C15—H15A	0.9600
C6—C7	1.378 (3)	C15—H15B	0.9600
C6—Cl1	1.7360 (19)	C15—H15C	0.9600
C7—H7	0.9300	N1—N2	1.3658 (18)
C8—O2	1.245 (2)	N2—H2	0.8600
C8—N2	1.357 (2)	O1—H1A	0.8200
C8—C9	1.461 (2)	O3—H3	0.8200
C9—C14	1.402 (2)	O4—H4A	0.8200
C9—C10	1.407 (2)	O5—H5A	0.8200
			
N1—C1—C2	118.17 (16)	O3—C10—C9	121.19 (15)
N1—C1—H1	120.9	C11—C10—C9	121.57 (16)
C2—C1—H1	120.9	C10—C11—C12	119.67 (16)
C7—C2—C3	118.76 (16)	C10—C11—H11	120.2
C7—C2—C1	119.42 (16)	C12—C11—H11	120.2
C3—C2—C1	121.83 (16)	O4—C12—C11	116.74 (16)
O1—C3—C4	118.19 (16)	O4—C12—C13	122.75 (16)
O1—C3—C2	121.47 (16)	C11—C12—C13	120.51 (16)
C4—C3—C2	120.34 (17)	C14—C13—C12	119.19 (16)
C5—C4—C3	119.95 (18)	C14—C13—H13	120.4
C5—C4—H4	120.0	C12—C13—H13	120.4
C3—C4—H4	120.0	C13—C14—C9	122.15 (16)
C6—C5—C4	120.20 (18)	C13—C14—H14	118.9
C6—C5—H5	119.9	C9—C14—H14	118.9
C4—C5—H5	119.9	O5—C15—H15A	109.5
C5—C6—C7	120.58 (18)	O5—C15—H15B	109.5
C5—C6—Cl1	120.16 (15)	H15A—C15—H15B	109.5
C7—C6—Cl1	119.26 (16)	O5—C15—H15C	109.5
C6—C7—C2	120.17 (18)	H15A—C15—H15C	109.5
C6—C7—H7	119.9	H15B—C15—H15C	109.5
C2—C7—H7	119.9	C1—N1—N2	120.53 (15)
O2—C8—N2	119.10 (15)	C8—N2—N1	116.21 (14)
O2—C8—C9	121.77 (15)	C8—N2—H2	121.9
N2—C8—C9	119.14 (15)	N1—N2—H2	121.9
C14—C9—C10	116.89 (15)	C3—O1—H1A	109.5
C14—C9—C8	124.50 (15)	C10—O3—H3	109.5
C10—C9—C8	118.58 (15)	C12—O4—H4A	109.5
O3—C10—C11	117.23 (15)	C15—O5—H5A	109.5
			
N1—C1—C2—C7	−176.71 (16)	N2—C8—C9—C10	−177.89 (15)
N1—C1—C2—C3	3.2 (3)	C14—C9—C10—O3	178.17 (16)
C7—C2—C3—O1	−179.78 (16)	C8—C9—C10—O3	0.2 (2)
C1—C2—C3—O1	0.4 (3)	C14—C9—C10—C11	−0.9 (3)
C7—C2—C3—C4	0.1 (3)	C8—C9—C10—C11	−178.90 (16)
C1—C2—C3—C4	−179.76 (16)	O3—C10—C11—C12	−177.66 (17)
O1—C3—C4—C5	−179.61 (18)	C9—C10—C11—C12	1.5 (3)
C2—C3—C4—C5	0.5 (3)	C10—C11—C12—O4	179.64 (17)
C3—C4—C5—C6	−0.6 (3)	C10—C11—C12—C13	−0.8 (3)
C4—C5—C6—C7	0.1 (3)	O4—C12—C13—C14	179.18 (18)
C4—C5—C6—Cl1	179.57 (16)	C11—C12—C13—C14	−0.3 (3)
C5—C6—C7—C2	0.5 (3)	C12—C13—C14—C9	0.9 (3)
Cl1—C6—C7—C2	−178.96 (13)	C10—C9—C14—C13	−0.3 (3)
C3—C2—C7—C6	−0.6 (3)	C8—C9—C14—C13	177.58 (17)
C1—C2—C7—C6	179.28 (16)	C2—C1—N1—N2	−178.63 (14)
O2—C8—C9—C14	−175.36 (17)	O2—C8—N2—N1	5.6 (2)
N2—C8—C9—C14	4.3 (3)	C9—C8—N2—N1	−174.10 (14)
O2—C8—C9—C10	2.5 (3)	C1—N1—N2—C8	177.27 (15)

### Hydrogen-bond geometry (Å, º)

**Table:**

*D*—H···*A*	*D*—H	H···*A*	*D*···*A*	*D*—H···*A*
N2—H2···O5	0.86	2.01	2.833 (2)	159
O1—H1*A*···N1	0.82	1.81	2.5333 (19)	146
O3—H3···O2	0.82	1.77	2.5030 (18)	148
O4—H4*A*···O1^i^	0.82	2.00	2.7401 (19)	151
O5—H5*A*···O3^i^	0.82	2.03	2.8346 (19)	168

Symmetry code: (i) −*x*+1, *y*+1/2, −*z*+1/2.
